# Skin Cell and Tissue Responses to Cross-Linked Hyaluronic Acid in Low-Grade Inflammatory Conditions

**DOI:** 10.1155/2023/3001080

**Published:** 2023-08-26

**Authors:** Benjamin Sanchez, Sandra Ferraro, Audrey Josset-Lamaugarny, Aurélie Pagnon, Charlie K. Hee, Lauren Nakab, Dominique Sigaudo-Roussel, Bérengère Fromy

**Affiliations:** ^1^Laboratoire Biologie Tissulaire et Ingénierie Thérapeutique, Centre national de la recherche scientifique (CNRS), UMR 5305, LBTI, 7 Passage du Vercors, F-69367 Lyon cedex 7, France; ^2^University of Lyon 1, UMR 5305, LBTI, 7 Passage du Vercors, F-69367 Lyon cedex 7, France; ^3^NOVOTEC, ZAC du Chêne Europarc, 11 Rue Edison, 69500 Bron, France; ^4^Allergan Aesthetics, An AbbVie Company, 2525 Dupont Dr., Irvine, CA 92612, USA

## Abstract

Hyaluronic acid (HA), used in a variety of medical applications, is associated in rare instances to long-term adverse effects. Although the aetiology of these events is unknown, a number of hypotheses have been proposed, including low molecular weight of HA (LMW-HA) in the filler products. We hypothesized that cross-linked HA and its degradation products, in a low-grade inflammatory microenvironment, could impact immune responses that could affect cell behaviours in the dermis. Using two different cross-linking technologies VYC-15L and HYC-24L+, and their hyaluronidase-induced degradation products, we observed for nondegraded HA, VYC-15L and HYC-24L+, a moderate and transient increase in IL-1*β*, TNF-*α* in M1 macrophages under low-grade inflammatory conditions. Endothelial cells and fibroblasts were preconditioned using inflammatory medium produced by M1 macrophages. 24 h after LMW-HA fragments and HA stimulation, no cytokine was released in these preconditioned cells. To further characterize HA responses, we used a novel *in vivo* murine model exhibiting a systemic low-grade inflammatory phenotype. The intradermal injection of VYC-15L and its degradation products induced an inflammation and cell infiltration into the skin that was more pronounced than those by HYC-24L+. This acute cutaneous inflammation was likely due to mechanical effects due to filler injection and tissue integration rather than its biological effects on inflammation. VYC-15L and its degradation product potentiated microvascular response to acetylcholine in the presence of a low-grade inflammation. The different responses with 2D cell models and mouse model using the two tested cross-linking HA technologies showed the importance to use integrative complex model to better understand the effects of HA products according to inflammatory state.

## 1. Introduction

Hyaluronic acid (HA), a major glycosaminoglycan of the extracellular matrix, plays an important role in a number of biological processes such as wound healing, cancer, or embryonic development [1]. Cross-linked HA is used in a variety of medical applications, including as a dermal filler in aesthetics applications. Although typically regarded as an inert, biocompatible material due to its similarity with native HA, there have been rare instances of adverse effects which can occur from 4 weeks to more than 1 year posttreatment [2, 3]. Although the aetiology of these events is unknown, a number of hypotheses have been proposed, including biofilm-related response, a result of mechanical irritation with the product, or the presence of bacteria transferred into the skin during injection [4, 5]. They have also been proposed to be related to the low molecular weight of HA (LMW-HA) in the filler products, either as a component of the product or following its degradation [2, 5].

The size of HA fragments generated during degradation can also play a role in migration, apoptosis or immune regulation by activating the immune cells, such as macrophages and dendritic cell [6]. The high molecular weight polymers of HA (HMW-HA) (>500 kDa) are commonly described to have an anti-inflammatory effect, while low molecular weight polymers and fragments of HA (LMW-HA) (10 kDa to 500 kDa) and oligomers of HA (0.1 to 10 kDa) have been described as involved in inflammatory responses, possibly functioning as an alarm signal to the immune system, particularly for monocytes/macrophages [1, 7]. Moreover, the accumulation of LMW-HA seems to be strongly related to the severity of inflammatory pathologies and chronic diseases such as rheumatoid arthritis, liver injury, asthma and lung injuries, suggesting dysregulation in HA metabolism or accumulation due to an increase in HA catabolism with turnover of the matrix in these inflammatory diseases [8, 9]. LMW-HA have been widely described as proinflammatory [10–12] with some studies showing an increase in proinflammatory cytokine release, such as TNF-*α* and IL-1*β*, in the presence of LMW-HA [1, 10]. However, more recent studies have shown that the inflammatory responses observed on in vitro and ex vivo murine immune cells could be due to an endotoxin contamination on samples rather than the size of the fragments [13–15]. This discrepancy may be due to size-specific effects of the fragments or the culture conditions.

An additional factor that has been proposed in these events is an activation of the systemic immune response, like which occurs with flu, cold, or seasonal allergies [2, 5, 16]. Moreover, filler treatments are not recommended in case of active auto-immune diseases such as systemic lupus erythematosus, rheumatoid arthritis, or mixed connective tissue disease [3] that all share a low-grade inflammatory microenvironment [17]. This low-level inflammation may impact cell-cell interactions between immune cells, microvascular endothelial cells, and dermal fibroblasts leading to extracellular matrix alteration [18].

Based on clinical observations, practitioners are recommended to perform proper skin preparation prior to HA injections to avoid infection and to advise patients to delay filler treatment if areas of inflammation/infection are present [19]. Although the effects of HA on the inflammatory response have been examined, the effect of cross-linked hyaluronic acid and its degradation products on the skin, under low-level inflammatory conditions, remains unexplored. In the present study, we used two different cross-linking technologies VYC-15L (Vycross technology) and HYC-24L+ (Hylacross technology) and their hyaluronidase-induced degradation products [20, 21]. We hypothesized that a subclinical inflammation present at the time of the injection of degraded (HA fragments) and nondegraded cross-linked HA could impact immune responses that could affect cells found in the dermis. M1 macrophages and mature dendritic cells, both derived from monocytes THP-1 lineages (as immune cells models), inflammatory dermal fibroblasts and inflammatory human dermal microvascular endothelial cell (HDMEC) were used in vitro to study the effects of HA products and their LMW degradation fragments in the low-grade inflammatory environment. To further study in vivo the vascular and inflammatory responses, we used HA and their LMW degradation fragments in a systemic low-grade inflammatory murine model.

## 2. Materials and Methods

### 2.1. Hyaluronic Acid Degradation and Characterization

Hyaluronic acid VYC-15L (Juvéderm Volbella with Lidocaine) and HYC-24L+ (Juvéderm Ultra Plus XC) (Allergan Aesthetics, Pringy, France) both containing 0.3% Lidocaine were used. The final gel has a similar elasticity (G′) between VYC-15L (271 Pa) and HYC-24L+ (263 Pa). But, HYC-24L + has a higher HA concentration and cohesivity (24 mg/mL and 112 gmf) compared to VYC-15L (15 mg/mL and 19 gmf). They were degraded using the human recombinant hyaluronidase PH20 HYLENEX® (Halozyme Therapeutics, San Diego, California), for 6 hours at 150 UI/ml at 37°C. The enzyme was inactivated using silicon bath at 100°C for 10 min. A vehicle control solution was comprised of inactivated hyaluronidase. VYC-15L fragments and HYC-24L + fragments were analysed by steric exclusion chromatography (steric exclusion column: Shodex OHpak SB-806M-HQ reference F6429106 and a precolumn Shodex OHPak SB-G 6B reference F6709430) (HPLC with refractometer). The analyses were performed with an Alliance Waters 2690 HPLC and using a Dawn Heleos 2 Light Scattering Detector (MALS) from Wyatt Technology. Each analysis was carried out 3 times coming from different preparation of same lot.

### 2.2. Endotoxin Quantification

The vehicle control, VYC-15L fragments and HYC-24L + fragments were quantified by Endosafe PTS (Charles River). The Endosafe®-PTS (Portable Test System) is a rapid, point-of-use test system, which is comprised of a test cartridge along with a hand-held spectrophotometer. For this test, we used cartridge with 0.01 EU/ml sensitivity (PTS2001F). All experiments were carried out according to the manufacturer's instructions.

### 2.3. Macrophages M1 Polarization

THP-1 monocytes were incubated for 24 h at 5% CO_2_ 37°C with PMA (Phorbol 12-myristate 13-acetate from Sigma) to induce the differentiation into macrophages M0. Then macrophages M0 were polarized into proinflammatory M1 for 66 hours in the presence of 100 ng/ml of LPS, 100 ng/ml of PMA and 20 ng/ml of IFN-*γ* (Peprotech, France). Polarization of macrophages M1 were confirmed via cytokine profile (Supplementary Table S1 and Supplementary Figure S1) and were then put at rest for 24 h, as previously reported [22].

### 2.4. Mature Dendritic Cell (mDC) Differentiation

THP-1 monocytes were harvested by centrifugation, resuspended in serum-free culture medium (without 10% heat-inactivated FBS) at a concentration of 2 *∗* 105 cells/ml, into 6-well plates with 200 ng/ml of IL-4, 100 ng/ml of GM-CSF, 200 ng/ml of ionomycin (Sigma) and 20 ng/ml of TNF-*α* (Peprotech) and incubated for 4 days at 5% CO_2_ 37°C. Medium exchange was performed every 2 days with fresh cytokine-supplemented medium. After cell differentiation confirmed via cytokine profile (Supplementary Figure S2), cells were cultured with complete medium [23].

### 2.5. Human Cell Culture

M1 macrophages from THP-1 were cultured at 37°C 5% CO_2_ in RPMI1640 (Gibco Life Technologies) and supplemented with 10% heat-inactivated FBS, 4.5 g/L glucose (Sigma-Aldrich, Saint-Quentin-Fallavier), 1% penicillin/streptomycin 10 mM of HEPES, 1 mM sodium pyruvate and 0.05 mM of 2-mercaptoethanol (Gibco Life Technologies). Mature Dendritic cells (mDC) from THP-1 were cultured in RPMI1640 (Gibco Life Technologies) and supplemented with 10% heat-inactivated FBS, 4.5 g/L glucose (Sigma-Aldrich, Saint-Quentin-Fallavier), 1% penicillin/streptomycin 10 mM of HEPES and 1 mM sodium pyruvate in a 5% CO_2_ humidified incubator at 37°C. Human dermal fibroblasts were kindly provided by the Cell and Tissue Bank (Hôpital Edouard-Herriot, Lyon, France). Normal breast human skin tissue explants were obtained from the surgical discard of anonymous healthy patients who provided informed consent of adult donors in line with ethical guidelines (French Bioethics law of 2004). Cell strains were declared to the French Ministry of Research under the number DC-2008-162 [18]. Exclusion criteria were related to positive test result for hepatitis B or C, or HIV as well as obesity history. As previously stated [18], human dermal fibroblasts were cultured in a medium consisted in a ratio 1 :  1 of Dulbecco's modified Eagle's medium with Glutamax and F-12 medium (Invitrogen, Cergy Pontoise, France) supplemented with 10% FBS (Sigma-Aldrich, Saint-Quentin-Fallavier, France) and 1% penicillin/streptomycin (Gibco Life Technologies). Human dermal microvascular endothelial cells (HDMEC) from adult donors (Promocell Heidelberg, Germany) were cultured at 37°C 5% CO_2_ in the endothelial cell medium from Promocell composed by Dulbecco's modified Eagle's medium (DMEM) containing heparin 90 *μ*g/ml, hydrocortisone 1 *μ*g/ml supplemented with 2% Fetal Bovine Serum (FBS), 0.4% Endothelial Cell Growth Supplement (Promocell) and Epidermal Growth Factor (recombinant human) at 10 ng/ml [18]. Complete medium was supplemented with 1% penicillin/streptomycin (Gibco, Life Technologies, Carlsbad, CA, United States) at 37°C and 5% CO_2_. THP-1 cells, dermal fibroblasts and HDMEC were used below 20, 20 and 10 population doubling level, respectively.

### 2.6. Effect of HA and HA Degradation Products on Immune Cell Response

M1 macrophages and mDC were seeded at 200 000 cells/ml and stimulated with the vehicle control, VYC-15L fragments, VYC-15L, HYC-24L + fragments and HYC-24L + at 100 *µ*g/ml for 24 h, 48 h, and 72 h. LPS at 1 *µ*g/ml was included as a positive control. Cell supernatants were then collected and centrifuged at 10000 × g for 10 min at 4°C. Cytokines in M1 macrophage culture supernatant and mature DC (mDC) supernatant were quantified using Human Magnetic Luminex Assay from R&D Systems (Minneapolis, MN, United States) and the levels of IL-8 (CXCL8), TNF-*α*, and IL-1*β* were analysed using Bio-Plex MAGPIX Multiplex Reader and Bio-Plex Manager software from Bio-Rad (Bio-Rad Laboratory, Hercules, CA, United States). Results were obtained from 3 independent experiments.

### 2.7. Effect of HA and HA Degradation Products on Preconditioned Dermal Skin Cells

HDMEC and dermal fibroblasts were seeded at 50 000 cells/ml for 24 h. After cell adhesion, cells were preconditioned using inflammatory medium produced by M1 macrophages at 1/100 dilution factor for 48 h to induce a low-grade inflammatory phenotype as previously described [18]. The preconditioned cells were then washed twice with PBS or HEPES, depending on cell type, and stimulated with the Vehicle Control, VYC-15L fragments, VYC-15L, HYC-24L + fragments, or HYC-24L + at 100 *µ*g/ml for 72 h in order to evaluate HA effects in a long time period. LPS at 1 *µ*g/ml was included as a positive control. Results were obtained from 3 independent experiments from 3 donors for HDMEC and dermal fibroblasts. Cell supernatants were collected and centrifuged at 10000 × g for 10 min at 4°C. IL-8, IL-6, and IL-1*β* were quantified in dermal fibroblasts and HDMEC coculture supernatant using ELISA assay from Abcam. Preconditioned cells were compared to unstimulated cells (Ø).

### 2.8. Induction of a Systemic Low-Grade Inflammation (LGI) in Healthy Mice

Experiments were carried out in healthy male C57BL/6 mice (∼20 g body weight) from Charles River (Le Genest-St-Isle, France). Mice were housed with ad libitum access to food and water, in a regulated environment with a constant temperature of 24°C and a day/night cycle of 12 h/12 h. All experiments were conducted in accordance with European Union recommendations for animal experimentation and approved by Rhone-Alpes ethics committee and then by the Ministry of Research-Project Authorization Service (agreement #18529). Special effort was made to minimize the number as well as the stress and suffering of mice used in this study. Before experimentation, animals were acclimatized for 1 week. To induce a systemic low-grade inflammatory phenotype, 100 *µ*l of very low dose of LPS (O26 : B6) at 5 ng/kg or PBS (control) was injected by intraperitoneal injection (IP) 3 times per week for 6 weeks as previously described [24, 25]. In order to characterize the systemic low-grade inflammatory phenotype, blood was collected after sacrifice by intracardiac puncture and centrifuged at 3000 rpm during 10 min at 4°C to study CCL2 and IL-6 in the plasma using Luminex assay (R&D system). Cutaneous biopsy (punch 8 mm Ø at the back) was also performed for histological analysis and to study in the skin TNF-*α* and IL-1*β* gene expression using RT-qPCR and IL-6, IL-1*β*, COX-2, and VCAM-1 protein quantification using ELISA assay. These parameters collected from low-grade inflammatory mice were compared with those from healthy control mice. Acute intradermal injection (20 *µ*l) of LPS at 5 *µ*g/ml was used as a positive control to induce strong inflammation in healthy mice.

### 2.9. Assessment of Acetylcholine- (ACh-) Mediated Vasodilation

Two days prior to the microvascular experiments, the back was depilated with a depilatory lotion in mice under isoflurane to provide hairless areas for skin blood flow measurements and iontophoretic delivery of acetylcholine (ACh 2% Sigma) performed in normal and low-grade inflammatory mice.

One day prior to the microvascular experiments, normal and low-grade inflammatory mice received an acute intradermal injection (20 *µ*l at a concentration of 100 *µ*g/ml) of the Vehicle Control, VYC-15L fragments, VYC-15L, HYC-24L + fragments, or HYC-24L + undiluted.

For the microvascular experiments, all mice were anaesthetized with thiopental (65 mg·kg^−1^, i.p.) and placed in an incubator (MMS, Chelles, France) to maintain a stable skin temperature (35.0 ± 0.5°C). The cutaneous blood flow was measured using a laser Doppler probe (PF 4001 Master, Periflux, Perimed, Sweden) in response to iontophoretic delivery of ACh with an anodal current application (100 *μ*A for 10 s) at the site of intradermal injection. The laser Doppler signal expressed in arbitrary units (a.u.) was digitized with a 200 Hz sampling frequency using a computerized acquisition system (MP 100, Biopac System, CA). Local iontophoretic drug delivery was chosen to assess the in vivo cutaneous microvascular function while avoiding any systemic effects. Data collection started with a 1 minute control period before the ACh delivery and was continued for 20 minutes.

### 2.10. Mouse Skin Analysis

For histological analysis, mouse skin was taken from the lower back 24 h after an acute intradermal injection (20 *µ*l) of the Vehicle Control, VYC-15L fragments, VYC-15L, HYC-24L + fragments, or HYC-24L+. Skin biopsy was fixed in AFA solution (alcohol, formalin, and acetic acid). Samples were embedded in paraffin and sectioned at a thickness of 5 *µ*m. After dewaxing, the cuts were successively stained with Harris hematoxylin, eosin G, and saffron. The matrix elements were coloured in pinkish-yellow, the cytoplasm in pink, the nuclei in purple, and HA in dark violet.

Skin tissue lysate was analysed by ELISA assay on IL-6 from Abcam (Cambridge, UK). Skin tissue lysate was prepared by homogenization in T-PER lysis buffer and supplemented with protease and phosphatase inhibitor (Thermo Fisher Scientific) in TissueLyzer using magnetic beads. Skin tissue lysates were centrifuged 10 min at 10000*g* and 4°C. Supernatant were then collected and quantified.

### 2.11. Mouse RNA Isolation and Real-Time Quantitative PCR

Total RNA of skin samples was isolated using RNeasy Fibrous Tissue Mini Kit for skin biopsies (Qiagen, Courtaboeuf, France) according to the manufacturer's instructions. 500 ng of total RNA was reverse-transcribed into cDNA using PrimeScript^TM^ RT reagent kit (Takara, Shiga, Japan) and analysed by Real-Time QPCR using SYBR® Premix Ex Taq II (Takara) on an AriaMx Real-Time PCR system (Agilent Genomics, Santa Clara, CA, United States). All primers were provided by Eurogentec. RPL13A forward GATGGTGGTTCCTGCTGCCC and reverse GGCTTTCTCTTTCCTCCTCTCCTCC; ACTB forward CATTGCTGACAGGATGCAGAAGG and reverse TGCTGGAAGGTGGACAGTGAGG; TNF-*α* forward GGTGCCTATGTCTCAGCCTCTT and reverse GCCATAGAACTGATGAGAGGGAG and IL-1B forward TGGACCTTCCAGGATGAGGACA and reverse GTTCATCTCGGAGCCTGTAGTG. Results were normalized to RPL13A and ACTB. Relative quantification was calculated using the 2^ΔΔCt^ quantification method.

### 2.12. Statistical Analyses

The results are expressed as mean ± SD. ACh-mediated vasodilation was expressed as the percentage changes of the cutaneous blood flow over the basal values, calculated as the average over the 1 min control period before the iontophoretic delivery of ACh. Statistical analyses were performed in all obtained data using Prism (version 8.0, GraphPad Software Inc.). An unpaired *t*-test or one-way analysis of variance (ANOVA) followed by Dunnett's multiple comparison test was used to compare two or more independent groups, respectively. A *p* value less than 0.05 was regarded as statistically significant.

## 3. Results

### 3.1. HA Degradation Products: Characterization and Endotoxin Quantification

**Figure 1:**
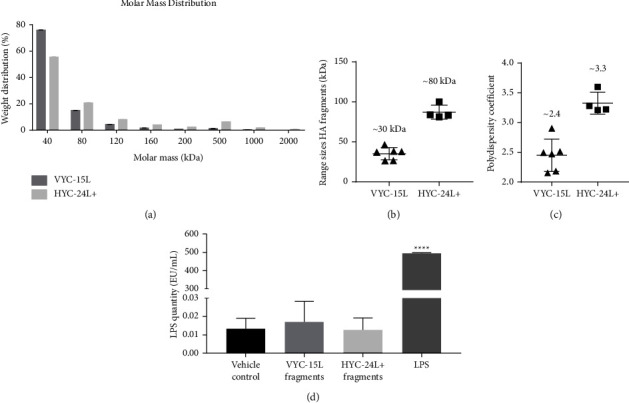
Characterization and endotoxin quantification of HA degradation products after a 6 hour incubation with hyaluronidase. Mean ± SD of weight distribution (%) in VYC-15L (*n* = 3) and HYC-24L+ (*n* = 3) (a). Scatter dot plot with mean ± SD of range sizes HA fragments (b) and polydispersity coefficient (c). Mean ± SD of LPS level (EU/ml) in the vehicle control (*n* = 2), VYC-15L fragments (*n* = 2), HYC-24L + fragments (*n* = 2), and LPS (positive control, *n* = 2) samples (d). ^*∗∗∗∗*^*p* < 0.0001 vs vehicle.

After a 6-hour incubation with hyaluronidase, VYC-15L and HYC-24L+ were degraded into fragments with a mean molecular weight of 30 ± 4.6 kDa and 80 ± 1.2 kDa and a polydispersity of 2.4 and 3.3, respectively. More than 75% of the total hyaluronidase-induced degradation products from VYC-15L were smaller than 40 kDa after 6 hours of degradation, while 55% of total HYC-24L + fragments were smaller than 40 kDa (Figure 1). Furthermore, degradation products from HYC-24L + contained more HMW fragments with a molecular weight >500 kDa than those from VYC-15L. These results indicate that HYC-24L+ is less degraded than VYC-15L for the same duration of degradation.

VYC-15L fragments, HYC-24L + fragments, and the Vehicle Control contained less than 0.02 EU/ml of LPS (Figure 1), showing that the degradation process did not introduce endotoxin and can be considered endotoxin-free in comparison to the positive LPS control (∼500 EU/ml).

### 3.2. Cytokines Release by M1 and mDC in Response to HA and HA Degradation Products

**Figure 2:**
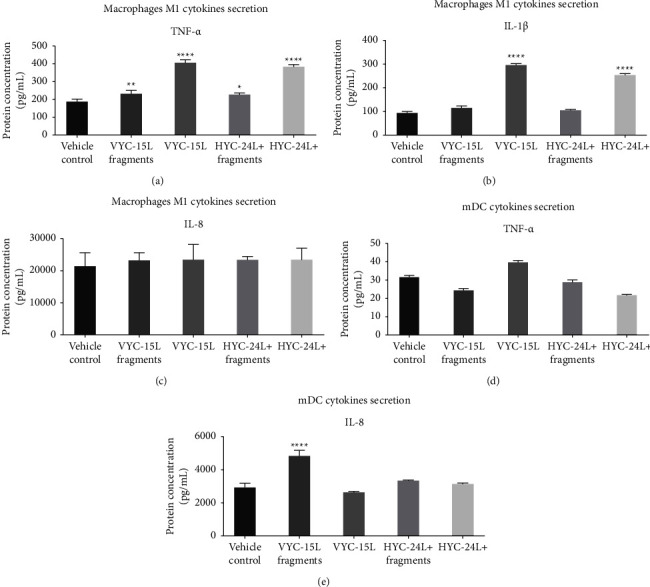
Effects of HA and HA degradation products on cytokines release by M1 (a–c) and mDC (d, e) at the 24 h time point. Mean ± SD of protein level (pg/ml) of TNF-*α* (a, d), IL-8 (c, e) and IL-1*β* (b), in the condition medium of M1 after stimulation with vehicle, VYC-15L fragments, VYC-15L, HYC-24L + fragments and HYC-24L+. ^*∗*^*p* < 0.01, ^*∗∗*^*p* < 0.001, ^*∗∗∗∗*^*p* < 0.0001 vs vehicle.

In the condition medium of M1 macrophages at the 24 h time point (Figure 2(a)), TNF-*α* protein was significantly overexpressed in the presence of VYC-15L fragments (233 ± 13 pg/ml), VYC-15L (407 ± 10 pg/ml), HYC-24L + fragments (227 ± 8 pg/ml), and HYC-24L + (384 ± 10 pg/ml) compared to the vehicle control (187 ± 5 pg/ml). IL-1*β* protein was only significantly overexpressed in the presence of VYC-15L (296 ± 9 pg/ml) and HYC-24L + (255 ± 5 pg/ml) compared to the vehicle control (94 ± 3 pg/ml) (Figure 2(b)). In contrast, IL-8 protein was not modified in any HA treatment compared to the vehicle control (21475 ± 390 pg/ml) (Figure 2(c)). However, the overexpression of cytokines by M1 mentioned above was much lower compared to the positive LPS control (Supplementary Figure S3A). There was no confounding effect of lidocaine component (data not shown).

In the condition medium of mDC at the 24 h time point (Figure 2(d)), no significant difference of TNF-*α* protein level was observed between conditions. Level of IL-1*β* was too low to be detectable by the Luminex assay except for LPS condition as positive control (Supplementary Figure S3B). IL-8 protein was only significantly overexpressed in the presence of VYC-15L fragments (4842 ± 325 pg/ml) compared to the vehicle control (2943 ± 242 pg/ml)(Figure 2(e)), although this overexpression was much lower compared to the positive LPS control (29697 ± 500 pg/ml) (Supplementary Figure S3B).

No differences were observed for any cytokines for either M1 macrophages or mDC, regardless of HA treatment group, at the 48 h and 72 h time points (Supplementary Figure S4), suggesting a transient effect of HA products in contrast to the positive LPS stimulation that lasted up to 72 h (data not shown).

### 3.3. Cytokines Released by Preconditioned Fibroblasts (Low-Grade Inflammatory Phenotype) in Response to HA and HA Degradation Products at 72 h

Compared to noninflammatory dermal fibroblasts, IL-6, IL-1*β,* and IL-8 were overexpressed in fibroblasts preconditioned with M1 macrophages but to a lesser extent than the positive LPS stimulation (Supplementary Figure S5), showing a low-grade inflammation in 72 h post-preconditioned fibroblasts.

**Figure 3:**
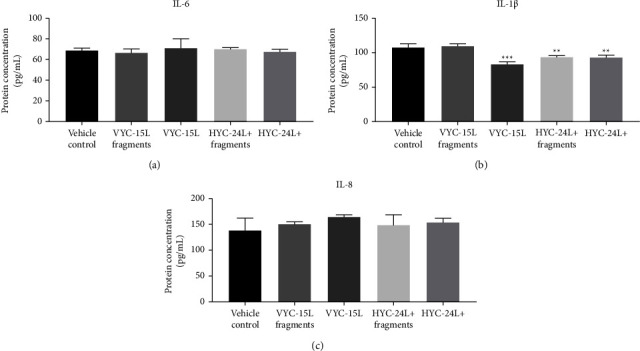
Effects of HA and HA degradation products on cytokines release by preconditioned dermal fibroblasts at the 72 h time point. Mean ± SD of protein level (pg/ml) of IL-6 (a), IL-1*β* (b), and IL-8 (c) in preconditioned dermal fibroblasts stimulated with vehicle, VYC-15L fragments, VYC-15L, HYC-24L + fragments, and HYC-24L+. ^*∗∗*^*p* < 0.01; ^*∗∗∗*^*p* < 0.001 vs the vehicle control.

In preconditioned dermal fibroblasts, VYC-15L fragments, VYC-15L, HYC-24L + fragments, and HYC-24L + did not induce overexpression of cytokines (IL-6, IL-1*β* and IL-8) compared to the vehicle control at 72 h (68 ± 2 pg/ml for IL-6; 105 ± 5 pg/ml for IL-1*β;* and 138 ± 17 pg/ml for IL-8) (Figure 3), except a slight downregulation of IL-1*β* (1.2-fold) in response to VYC-15L, HYC-24L + fragments and HYC-24L+. In contrast, the positive LPS stimulation induced a strong overexpression for IL-6 (503 ± 7 pg/ml), IL-1*β* (140 ± 5 pg/ml) and IL-8 (407 ± 9 pg/ml) in preconditioned dermal fibroblasts (Supplementary Figure S5).

### 3.4. Cytokines Released by Preconditioned HDMEC (Low-Grade Inflammatory Phenotype) in Response to HA and HA Degradation Products at 72 h

Compared to noninflammatory HDMEC, IL-8, IL-1*β,* and IL-6 were overexpressed in preconditioned HDMEC, but to a lesser extent than LPS stimulation (Supplementary Figure S6), showing a low-grade inflammation in 72 h post-preconditioned HDMEC.

**Figure 4:**
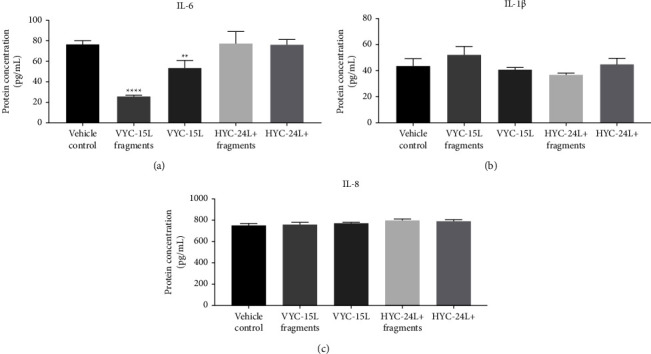
Effect of HA and HA degradation products at 72 h on cytokine release by preconditioned HDMEC. Mean ± SD of protein level (pg/ml) of IL-6 (a), IL-1*β* (b), and IL-8 (c) in preconditioned HDMEC stimulated with vehicle, VYC-15L fragments, VYC-15L, HYC-24L + fragments, and HYC-24L+. ^*∗∗*^*p* < 0.001, ^*∗∗∗∗*^*p* < 0.0001 vs the vehicle control.

In preconditioned HDMEC, VYC-15L fragments, VYC-15L, HYC-24L + fragments, and HYC-24L + did not induce overexpression of cytokines (IL-8 and IL-1*β*) compared to the vehicle control at 72 h (756 ± 6.9 pg/ml for IL-8 and 44 ± 3.9 pg/ml for IL-1*β*) (Figure 4). However, IL-6 expression (Figure 4(a)) decreased in the presence of VYC-15L fragments (25 ± 2 pg/ml) and VYC-15L (53 ± 6 pg/ml) compared to the vehicle control (76 ± 3 pg/ml). In contrast, the positive LPS stimulation induced a strong overexpression for IL-8 (794 ± 13 pg/ml), IL-1*β* (76 ± 4 pg/ml), and IL-6 (792 ± 15 pg/ml) in preconditioned HDMEC (Supplementary Figure S6).

### 3.5. In Vivo Mouse Model of Systemic Low-Grade Inflammation

The plasma level of IL-6 was higher in systemic low-grade inflammatory mice (19 ± 4 pg/ml) than in control mice (8 ± 1 pg/ml), representing an increase of 225%. Similarly, CCL2 plasma level was higher in low-grade inflammatory mice (239 ± 50 pg/ml) than in control mice (148 ± 43 pg/ml), representing an increase of 161% (Supplementary Figure S7A).

In the skin, the upregulations of TNF-*α* (2.8-fold) and IL-1*β* (1.3-fold) gene expression in the skin of systemic low-grade inflammatory mice were not significant compared to control mice (Supplementary Figure S7B). Similarly, the increase of +130% in COX-2 protein concentration in the skin of systemic low-grade inflammatory mice (2410 ± 385 pg/ml) was not significant compared to control mice (1868 ± 312 pg/ml), such as LPS positive control (1764 ± 256 pg/ml) (Supplementary Figure S7C). No increase of IL-6 protein concentration in the skin of systemic low-grade inflammatory mice (103.9 ± 22 pg/ml) was observed compared to control mice (102 ± 16 pg/ml) (Supplementary Figure S7C). Again, the increase of +123% in VCAM-1 protein concentration in the skin of systemic low-grade inflammatory mice (8493 ± 1418 pg/ml) was not significant compared to control mice (6903 ± 1695 pg/ml) (Supplementary Figure S7C). Systemic low-grade inflammation increased intragroup heterogeneity as shown by the increased coefficient of variation (CV) in low-grade inflammatory mice compared to control mice: 21% vs 7% for IL-6 plasma level, 29% vs 20% for CCL2 plasma level, 47% vs 39% for TNF-*α* gene expression, and 103% vs 71% for IL-1*β* gene expression and correlated with standard deviation observed. Thus, the cytokine expression was increased in the plasma (systemic inflammation), and there was no overexpression, only trends with a high variability, in the skin. These moderate increases in cytokine expression were expected to obtain a mouse model of systemic low-grade inflammation in the skin, and not strong inflammation obtained in the positive control mice (Supplementary Figure S7). In addition, no differences were observed between control mice and systemic low-grade inflammatory mice on skin structure by histological analysis. No higher immune cell infiltration and differences in thickness were observed in comparison to the infiltration observed in the positive control mice (Supplementary Figure S7D).

On the vascular aspect, basal skin blood flow was not different between control mice (45 ± 13 arbitrary units (a.u.)), systemic low-grade inflammatory mice (49 ± 11 a.u.), and positive control mice (49 ± 9 a.u.). Similarly, the ACh-mediated vasodilation was not different between systemic low-grade inflammatory mice (32 ± 15%) and control mice (43 ± 12%), in contrast to the augmented ACh-mediated microvascular reactivity in the positive control mice (69 ± 32%) (Supplementary Figure S7E).

### 3.6. Effects of HA and HA Degradation Products on Skin Structure in Mice

**Figure 5:**
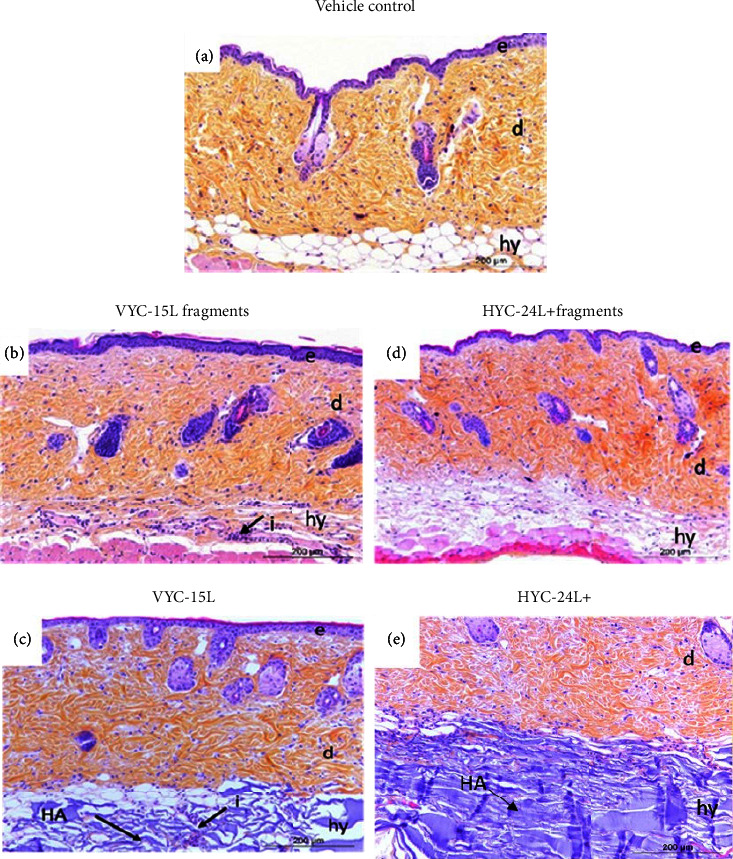
Representative HES coloration skin (×20) in systemic low-grade inflammatory mice 24 h after an acute intradermal injection (20 *µ*l) of the vehicle control (a), VYC-15L fragments (b), VYC-15L (c), HYC-24L + fragments (d), and HYC-24L+ (e). *e* = epidermis, *d* = dermis, hy = hypodermis, *i* = inflammation, and HA = hyaluronic acid. Hyaluronic acid is colored in dark violet, extracellular elements in yellow, and nuclei of cells in purple.

To study the effects of HA and HA degradation products in a systemic low-grade inflammation environment, the histological analyses were performed 24 h after the intradermal injections of vehicle, VYC-15L fragments, VYC-15L, HYC-24L + fragments, and HYC-24L+ in systemic low-grade inflammatory mice (Figure 5).

Vehicle induced a very limited inflammation (Figure 5(a)). The injection of VYC-15L fragments and VYC-15L induced a moderate inflammation around the products (Figures 5(b) and 5(c)) with immune cell recruitment. While, in the presence of HYC-24L + products (Figures 5(d) and 5(e)) immune cells seemed to be less infiltrated than in the presence of VYC-15L products. Due to higher degradation of VYC-15L than HYC-24L+ (Figure 1), VYC-15L fragments were more difficult to observe than HYC-24L fragments (Supplementary Figure S8).

### 3.7. Skin IL-6 and IL-1*β* in Response to HA and HA Degradation Products in Control and Systemic Low-Grade Inflammatory Mice

**Figure 6:**
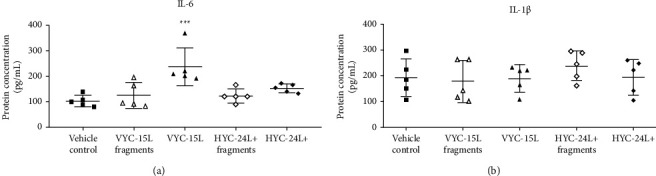
Scatter dot plot IL-6 (a) and IL-1*β* (b) protein concentration. Mean ± SD of protein level (pg/ml) in skin in systemic low-grade inflammatory mice (*n* = 5), 24 h after an acute intradermal injection (20 *µ*l) of the vehicle control, VYC-15L fragments, VYC-15L, HYC-24L + fragments, and HYC-24L+. All samples were normalized to 1000 *µ*g of total protein. ^*∗∗∗*^*p* < 0.001 vs the vehicle control.

In systemic low-grade inflammatory mice, IL-6 protein in the skin was only significantly overexpressed at 24 h in the presence of VYC-15L (238 ± 52 pg/ml) compared to the vehicle control mice (102 ± 20 pg/ml) (Figure 6), associated with an enhanced intragroup heterogeneity. In contrast, no differences were observed for the IL-1*β* protein concentration in the skin between each condition at 24 h.

### 3.8. Effects of HA and HA Degradation Products on Skin Microcirculation in Mice

**Figure 7:**
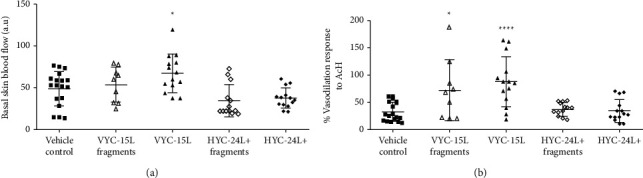
Scatter dot plot (mean ± SD) basal skin blood flow (a) and ACh-mediated vasodilation (b) in systemic low-grade inflammatory (*n* = 5) mice 24 h after intradermal injection of the vehicle control, VYC-15L fragments, VYC-15L, HYC-24L + fragments, and HYC-24L+. ^*∗*^*p* < 0.05, ^*∗∗∗∗*^*p* < 0.0001 vs the vehicle control. Each point represents one measurement of microvascular experiments.

In systemic low-grade inflammatory mice, basal skin blood flow was increased by the injection of VYC-15L (67 ± 18 a.u.) compared to the vehicle control (49 ± 11 a.u.) (Figure 7(a)), although this significant increase was slight. ACh-mediated vasodilation was significantly increased by the injection of VYC-15L (87 ± 33%) and VYC-15L fragments (72 ± 40%) compared to the vehicle control (32 ± 15%) (Figure 7(b)). This enhanced ACh-mediated vasodilation was associated with an elevated heterogeneity as shown by an increased standard deviation.

## 4. Discussion

Few fundamental studies have been done on delayed complications after intradermal HA injection. It has been hypothesized that HA degradation products may be related to the development of delayed complications. In this work, we hypothesized that an inflammatory state could be a key mechanism of skin complications in the presence of cross-linked HA. The objectives of this study were to assess the effect of degraded (HA fragments) and nondegraded cross-linked HA under systemic low-grade inflammatory conditions on immune and dermal cells as well as vascular responses.

For many years, concerns regarding pro- and anti-inflammatory effects of HA fragments have been raised [13]. LMW-HA fragments were described as a danger proinflammatory signal, which stimulates stromal fibroblasts to perform an immune response and to provide a favourable environment for infiltrating immune cells [26–28]. Indeed, LMW-HA are described as an active proinflammatory molecule stimulating particularly innate immune cells, monocytes and macrophages, to produce a palette of proinflammatory cytokines and chemokines [29]. Release of these cytokines recruits neutrophils and drives T lymphocyte differentiation [7, 10, 30]. However, it was later shown that the proinflammatory response related to LMW-HA fragments and HA could be due to endotoxin contamination from the sources of HA, enzyme, and methods for degradation [13]. In the current study, we showed that all the HA fragments and HA used were endotoxin-free (at least below detection threshold), excluding any confounding effects.

Hyaluronic acid has several functions depending on its molecular weight and several studies reported HA inflammatory modulation in a size-specific manner. In the present study, two sizes of HA fragments were obtained for different filler formulations, 30 kDa and 80 kDa, under the same experimental conditions, highlighting potential differences between the two HA fillers, VYC-15L, and HYC-24L+, respectively. The difference of size between VYC-15L and HYC-24L + fragments may be related to the composition or cross-linking technology of each filler or to their initial HA concentration, 15 mg/ml or 24 mg/ml for VYC-15L and HYC-24L+, respectively [20, 31, 32]. Both are cross-linked with 1,4-butanediol diglycidyl ether (BDDE), but the composition of the material (Hylacross is HMW and Vycross is HMW and LMW) could allow for minor differences in the final rheological properties [32, 33]. The LMW-HA in VYC-15L allows for the chains to be closer together at cross-linking, which results in both a tighter and more efficient cross-linking. Interestingly, we degraded the two HA fillers at different time point inducing different range sizes of LMW-HA fragments and no inflammatory effect was observed overtime in nonpreconditioned cells (data not shown), correlated with previous studies using endotoxin-free enzyme [13, 14].

We observed for nondegraded HA, VYC-15L, and HYC-24L+, a moderate and transient increase in IL-1*β*, TNF-*α* in M1, but no change in cytokine release in inflammatory fibroblasts and inflammatory endothelial cells; contrary to LPS stimulation inducing a strong inflammatory response on immune cells and dermal skin cells.

About immune cells, activated M1 responded to VYC-15L and HYC-24L + by further elevating the production of TNF-*α* and IL-1*β*, whereas only TNF-*α* was further increased with fragments of VYC-15L and HYC-24L + at 24 h. The majority of immune cells do not bind HA until they are activated by an antigenic or inflammatory agent [13, 34, 35]. In the present study, CD44 gene expression was highly increased in M1 and dendritic cells compared to M0 and THP-1, suggesting a HA binding on M1 with the production of TNF-*α* and IL-1*β* [34, 36]. TNF-*α* level seems to play a major role in the immune HA modulation. In the current study, the lower production or TNF-*α* observed in response to fragments from both HA fillers compared to their respective nondegraded HA could reveal an alteration of HA fragments binding affinity or that there was no inflammatory effect due to HA fragments, supported by no further production of IL-1*β*. Indeed, CD44-mediated HA effects are more complex in terms of receptor density, affinity to HA and kinetic of response [35, 36].

The results obtained underline the main role of activated macrophages at 24 h that was not persistent at 48 h and 72 h, indicating a transient transmission of inflammatory cytokines in the presence of degraded and nondegraded HA, without compensative effects of anti-inflammatory factors. In contrast, direct LPS stimulation on M1 and mDC induced a strong and sustained inflammation up to 72 h balanced by the release of anti-inflammatory cytokines, contrasting with the mild response obtained with nondegraded and degraded HA products (Supplementary Figure S3).

These transient and moderate inflammatory responses on immune cells highlighted that HA fragments did not induce inflammation over time, but suggest a possible cell communication with other dermal skin cells such as dermal fibroblasts and microvascular endothelial cells. Indeed, an important outcome of studies on molecular mechanisms that underlie inflammation was that an inflammatory response is not a specialized function of cells of hematopoietic origin (macrophages, neutrophils, lymphocytes etc.) but rather a fundamental attribute of several cellular types. In this context, fibroblasts play a critical role in modulating immune cells such as leukocytes and could be responsible for the establishment of chronic inflammation [37]. In addition, endothelial cells synthesize and secrete chemokine and cytokines, which are implicated in recruiting immune cells in response to inflammatory disorders [38]. This inside-out inflammatory response [18] has been supplemented with an outside-in signalling involving activated proinflammatory fibroblasts interacting with immune cells to direct endothelial activation [39]. In the present study, although endothelial cells and fibroblasts under proinflammatory M1 stimulation exhibited proinflammatory phenotype by releasing increased levels of IL-1*β*, IL-8, and IL-6 no major change was observed for both cell types in the presence of degraded or nondegraded HA.

Altogether, these in vitro experiments showed that HA fragments in an inflammatory environment did not induce strong inflammatory responses over time, while nondegraded HA induced transient inflammatory modulation.

To better characterize the cellular response observed after cross-linked HA injection [1, 4], we also used an in vivo murine model. It appeared that indirect coculture model present limitation and cannot reflect complexity of the skin whose homeostasis relies on a finely tuned equilibrium of well-regulated interactions between the different layers of the skin with their cellular and subcellular structures [40]. Very low dose endotoxin in the blood circulation is one of the emerging risk factors that can cause chronic low-grade inflammation in humans and animals [41, 42]. In this way, to mimic a systemic low-grade inflammatory phenotype on mice, repeated intraperitoneal injections of a very low dose of LPS for 6 weeks in healthy mice were carried out, based on previous studies [24, 25]. This chronic LPS treatment increased proinflammatory cytokines (IL-6 and CCL2) in the plasma, with no significant skin inflammation although inflammatory markers (TNF-*α*, IL-1*β,* and COX-2) revealed a trend to increase within the skin. These characteristics are concordant with skin weakened by systemic low-grade inflammation described in chronic pathologies such as obesity and aging [43, 44] and validating our mouse model of low-grade inflammation. This in vivo mouse model of systemic low-grade inflammation was then used to study the effects induced by intradermal injections of HA and its degradation products and evaluate differences between the two cross-linking technologies on skin integrity and microvascular reactivity.

Histological analysis revealed intact skin tissue architecture around the injection site in all mice that received vehicle in the presence of systemic low-grade inflammation, revealing the nontraumatic nature of the intradermal injection in healthy adult mice. Regarding the injection of exogenous HA and its degradation products, we showed an acute inflammation and cell infiltration into the skin more pronounced with VYC-15L compared to HYC-24L+, in systemic low-grade inflammatory mice. Likewise, nondegraded VYC-15L was the only product to significantly increase the IL-6 concentration in the skin in the presence of systemic low-grade inflammation (+231%), confirming the results highlighted by histological analyses. In addition, IL-1*β* was not oversecreted in the presence of VYC-15L, suggesting that the common inflammatory pathway described in literature using CD44 was not involved in the inflammatory response to the presence of VYC-15L [9]. We suggest that this cutaneous inflammation induced by VYC-15L injection was likely due to mechanical effects due to filler injection and tissue integration rather than its biological effects, since degradation products did not increase IL-6 protein concentration. In contrast, HYC-24L + did not modulate IL-6 concentration in the skin. These different observations between VYC-15L and HYC-24L + may be related to their different rheological properties, degree of cross-linking and concentration of HA inside the gel that may have different biological and physical effects [20, 32, 45, 46]. Furthermore, contrary to the literature we observed no anti-inflammatory effect in the presence of nondegraded HA, suggesting that these biological effects are dependent to the structure of HA and to tissue localization, if its native or cross-linked HA [47, 48].

The use of acetylcholine (ACh) is the gold standard method to assess the vascular function that relies on vasomotor factors present in the tissue microenvironment. Normal vascular response to ACh relies mostly on endothelial L-arginine/nitric oxide (NO) pathway, which can be altered in pathological condition with inflammation features leading to a decreased involvement of the NO pathway during ageing, diabetes [49, 50]. However, in a low-grade inflammatory pathology such as obesity [43], an increase in Ach-induced vasodilation response has been reported to be promoted by COX pathway. We highlighted that VYC-15L was the only product capable of significantly modulating the microvascular reactivity of the skin in the presence of a systemic low-grade inflammation. Indeed, basal vascular tone of the skin was not changed by the injection of HA and their degradation products in all mice, except a slight increase following the VYC-15L injection. In addition, we showed that endothelium-dependent vasodilations in response to ACh were increased by VYC-15L and its degradation product compared to the vehicle control. This potentiated acetylcholine response, which was not observed with HYC-24L+, was similar to the positive LPS control mice exhibiting a strong inflammation. Surrogate signaling pathways including inflammatory factors such as COX-2 can be involved in vascular ACh sensitization in low-grade inflammatory pathology [43, 51]. We thus quantified the amount of VCAM-1 which is a marker for vessels integrity and COX-2 as vasodilator mediators [50, 52, 53]. Since these markers were not overexpressed either in the presence of a systemic low-grade inflammation or in the presence of VYC-15L, we suggest that this vascular modulation involved another pathways or mechanisms, which require further study. Altogether, these short-term in vivo experiments using an inflammatory integrative murine model induced by a very low dose of LPS, have shown that intradermal injection of nondegraded VYC-15L products increase IL-6 in the skin, cells infiltration, skin perfusion, and microvascular sensitivity in response to ACh, showing a moderate and transient cutaneous proinflammatory response in contrast to HYL-24L+.

## 5. Conclusion

Any inflammation that was observed in the present study was transient and independent of the degradation (i.e., HA fragments were not shown to impact the inflammatory response) and may be more related to physical properties than biologic stimulation. We could suggest that differences in rheological properties or cross-linking technologies may interact with skin components and actors, leading to different responses to VYC-15L in systemic low-grade inflammatory conditions.

In addition, an in vivo model of low-level inflammation was developed to evaluate a change in the basal state and its impact on inflammatory responses to HA and its degraded products. Some changes were observed with VYC-15L, independent of degradation state, as it relates to short-term inflammatory response and vascular sensitivity. This vascular sensitivity could be linked to the macrophage-mediated inflammation related to an increased recruitment of immune cells at the injection site and to the transient inflammatory modulation observed in 2D models. This suggests a potential acute response, although it is unclear whether this would be related to long-term events or just short-term tissue response. Further studies would be needed to clarify the potential link with the reported rate of adverse reactions to the two types of cross-linked hyaluronan and does this correlate with our results. From the perspective of a practicing dermatologist or other aesthetic physician, a proinflammatory profile prior to HA injection may be necessary to inform about possible side effects, as recently mentioned by Rivers (19), in particular for VYC-15L based on our results.

The different responses between 2D cell models and mouse model showed the importance to use integrative complex model to better understand the effects of HA products according to inflammatory state.

## Data Availability

The data used to support the findings of this study are available from the corresponding author upon request. The data are not publicly available due to ethical restrictions.

## Supplementary Materials

Supplementary Materials Supplementary Table S1: characterization of the proinflammatory M1 culture medium. Supplementary Figure S1: proinflammatory macrophages M1 phenotypes. Mean ± SD of M1 markers gene expression of TNF-*α*, IL-6, IL-8, IL-1*β*, CD64, CD68, and CD44. Genes expressions are normalized with GAPDH and TBP and data are represented in fold change. ^*∗*^*p* < 0.05, ^*∗∗*^*p* < 0.01, ^*∗∗∗*^*p* < 0.001, ^*∗∗∗∗*^*p* < 0.0001 vs macrophages M0. Data are representative of at least three independent experiments. Supplementary Figure S2: proinflammatory mature dendritic cell (mDC) phenotype. Mean ± SD of mDC markers gene expression of TNF-*α*, IL-6, IL-8, IL-1*β*, CD206, TGM2, and CD44. Genes expressions are normalized with GAPDH and TBP and data are represented in fold change. ^*∗*^*p* < 0.05, ^*∗∗*^*p* < 0.01, ^*∗∗∗*^*p* < 0.001 vs THP-1. Data are representative of at least three independent experiments. Supplementary Figure S3: LPS effect on M1 and mDC cytokine production at 24 h. Supernatant produced by immune cells. Mean ± SD of protein level (pg/ml) of TNF-*α*, IL-1*β,* and IL-8 in macrophages M1 (A) and mDC (B) after 24 h stimulation of LPS at 1 *µ*g/ml. Data are representative of at least three independent experiments. ^*∗∗∗∗*^*p* < 0.0001 vs unstimulated (Ø) immune cells. Supplementary Figure S4: Effects of HA and HA degradation products on cytokines release by M1 and mDC at the 24 h, 48 h, and 72 h time points. Mean ± SD of protein level (pg/ml) of TNF-*α*, IL-8, and IL-1*β* (except for mDC), in the condition medium of M1 (A) and mDC (B) after stimulation with vehicle, VYC-15L fragments, VYC-15L, HYC-24L + fragments, and HYC-24L+. For each condition, left column corresponds to 24 h, middle column corresponds to 48 h, and right column corresponds to 72 h stimulation. Data are representative of at least three independent experiments. ^*∗∗*^*p* < 0.01, ^*∗∗∗∗*^*p* < 0.0001 vs the respective vehicle control. Supplementary figure S5: low-grade inflammation phenotype for preconditioned dermal fibroblasts. Cytokine release in unstimulated (Ø), preconditioned (PC), and LPS-stimulated dermal fibroblasts. Mean ± SD of protein level (pg/ml) of IL-8 (A), IL-1*β* (B) and IL-6 (C) 72 h post preconditioning. LPS is considered as the positive control for inflammation. ^*∗∗∗*^*p* < 0.001, ^*∗∗∗∗*^*p* < 0.0001. vs nonpreconditioned (noninflammatory) unstimulated fibroblast (Ø). Supplementary Figure S6: low-grade inflammation phenotype for preconditioned HDMEC. Cytokine release in unstimulated (Ø), preconditioned (PC) and LPS-stimulated dermal endothelial cells (HDMEC). Mean ± SD of protein level (pg/ml) of IL-8 (A), IL-1*β* (B), and IL-6 (C) 72 h post preconditioning. LPS is considered as positive control for inflammation. ^*∗∗∗∗*^*p* < 0.00001 vs nonpreconditioned (noninflammatory) unstimulated HDMEC (Ø). Supplementary Figure S7: phenotype of systemic low-grade inflammation induced by a chronic treatment of low dose of LPS in mice. Scatter dot plot showing: A, IL-6 and CCL2 plasma level in control mice (*n* = 4) and in systemic low-grade inflammatory (LGI) mice (*n* = 8); B, fold change gene expression of TNF-*α* and IL-1*β* in skin biopsy from control mice (*n* = 5), LGI mice (*n* = 15) and positive control mice treated with an acute intradermal injection of LPS (*n* = 5); *C*, protein level of IL-6, IL-1*β*, COX-2 and VCAM-1 in control mice (*n* = 5), LGI mice (*n* = 5) and positive LPS control mice (*n* = 5); *D*, representative histological analysis with HES coloration skin in one control mouse, one LGI mouse, and one positive LPS control mice; *E*, vascular properties with basal skin blood flow and acetylcholine (ach)-mediated microvascular reactivity in control mice (*n* = 17), LGI mice (*n* = 18), and positive LPS control mice (*n* = 15). ^*∗*^*p* < 0.05, ^*∗∗*^*p* < 0.01, ^*∗∗∗*^*p* < 0.001, ^*∗∗∗∗*^*p* < 0.0001 vs control mice. Supplementary Figure S8: remained injected hyaluronan in the skin of low-grade inflammation (LGI) mice. Representative histological analysis with HES coloration skin in one LGI mouse 24 h after an acute intradermal injection (20 *µ*l) of VYC-15L fragments (A) and HYC-24L fragments (B). Numbers indicate locations with remained injected hyaluronan, which are shown with a larger magnification on the right-hand side.
