# Supplementary material for: Skin Cell and Tissue Responses to Cross-Linked Hyaluronic Acid in Low-Grade Inflammatory Conditions
Source: Int J Inflam. 2023 Aug 26;2023:3001080. doi: 10.1155/2023/3001080 (PMC10474960; doi:10.1155/2023/3001080)
Supplement: Supplementary Materials — Supplementary Table S1: characterization of the proinflammatory M1 culture medium. Supplementary Figure S1: proinflammatory macrophages M1 phenotypes. Mean ± SD of M1 markers gene expression of TNF-α, IL-6, IL-8, IL-1β, CD64, CD68, and CD44. Genes expressions are normalized with GAPDH and TBP and data are represented in fold change. ∗p < 0.05, ∗∗p < 0.01, ∗∗∗p < 0.001, ∗∗∗∗p < 0.0001 vs macrophages M0. Data are representative of at least three independent experiments. Supplementary Figure S2: proinflammatory mature dendritic cell (mDC) phenotype. Mean ± SD of mDC markers gene expression of TNF-α, IL-6, IL-8, IL-1β, CD206, TGM2, and CD44. Genes expressions are normalized with GAPDH and TBP and data are represented in fold change. ∗p < 0.05, ∗∗p < 0.01, ∗∗∗p < 0.001 vs THP-1. Data are representative of at least three independent experiments. Supplementary Figure S3: LPS effect on M1 and mDC cytokine production at 24 h. Supernatant produced by immune cells. Mean ± SD of protein level (pg/ml) of TNF-α, IL-1β, and IL-8 in macrophages M1 (A) and mDC (B) after 24 h stimulation of LPS at 1 µg/ml. Data are representative of at least three independent experiments. ∗∗∗∗p < 0.0001 vs unstimulated (Ø) immune cells. Supplementary Figure S4: Effects of HA and HA degradation products on cytokines release by M1 and mDC at the 24 h, 48 h, and 72 h time points. Mean ± SD of protein level (pg/ml) of TNF-α, IL-8, and IL-1β (except for mDC), in the condition medium of M1 (A) and mDC (B) after stimulation with vehicle, VYC-15L fragments, VYC-15L, HYC-24L + fragments, and HYC-24L+. For each condition, left column corresponds to 24 h, middle column corresponds to 48 h, and right column corresponds to 72 h stimulation. Data are representative of at least three independent experiments. ∗∗p < 0.01, ∗∗∗∗p < 0.0001 vs the respective vehicle control. Supplementary figure S5: low-grade inflammation phenotype for preconditioned dermal fibroblasts. Cytokine release in unstimulated (Ø), preconditione [file 3001080.f1.pdf]

Supplementary Materials

Supplementary Table S1: Characterization of the pro-inflammatory M1 culture medium

Cytokines	M0 (Mean pg/ml ± SD)	M1 (Mean pg/ml ± SD)	p-value
TNF-α	119±2	187±2	0.0211
IL-8	4151±51	21475±39	<0.0001
CCL2	198±14	213±11	ns
IL-1β	255±15	94±2	<0.0001
IFN-gamma	88±2	181±7	0.0014
CCL5/RANTES	202±7	410±8	<0.0001
GM-CSF	120±10	308±5	<0.0001
VEGF-A	72±2	147±2	<0.0001

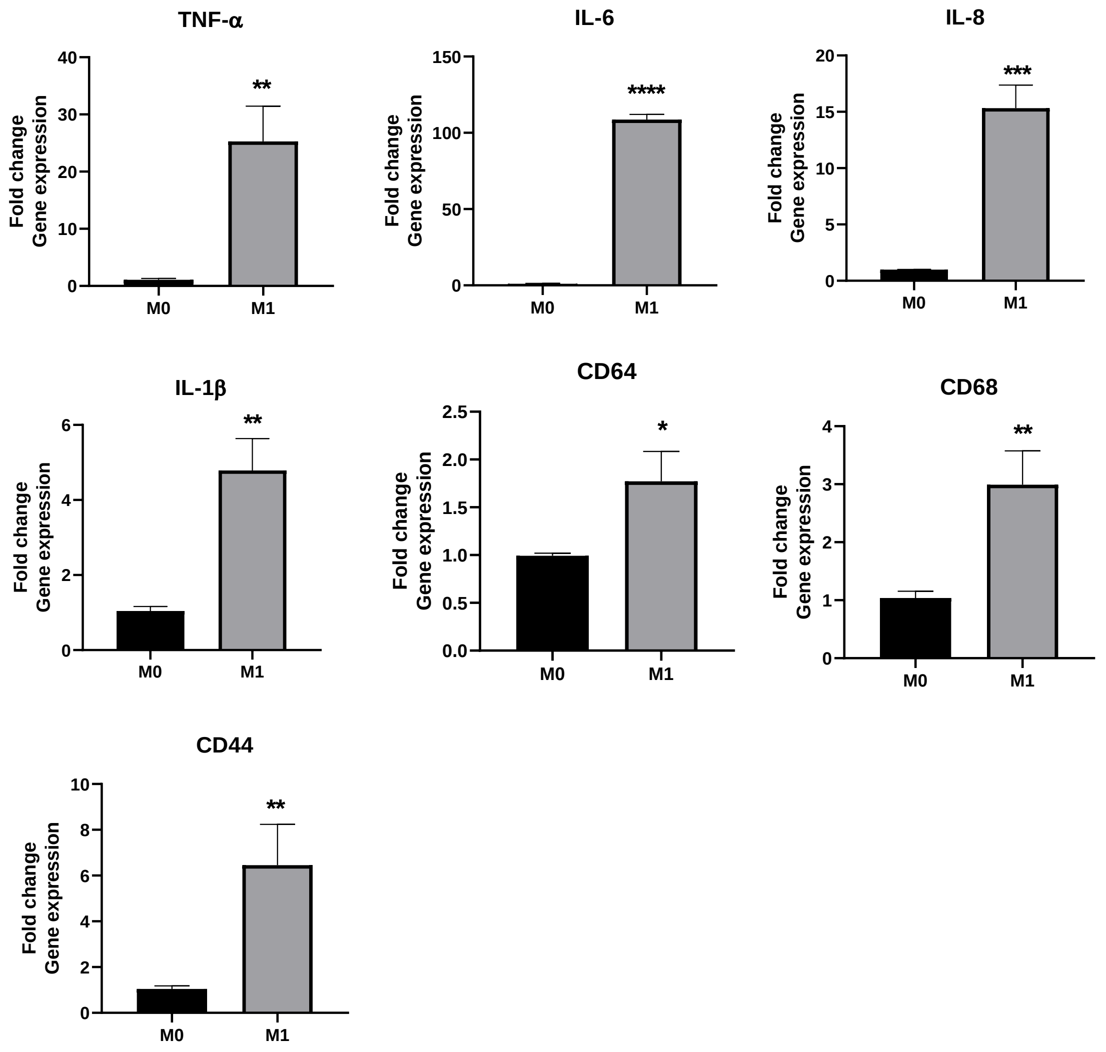

Supplementary Figure S1: Pro-inflammatory Macrophages M1 phenotypes. Mean±SD of M1 markers gene expression of TNF-α, IL-6, IL-8, IL1β, CD64, CD68 and CD44. Genes expressions are normalized with GAPDH and TBP and data are represented in fold change. *P<0.05, **P<0.01, ***P<0.001, ****P<0.0001 *vs* macrophages M0. Data are representative of at least three independent experiments.

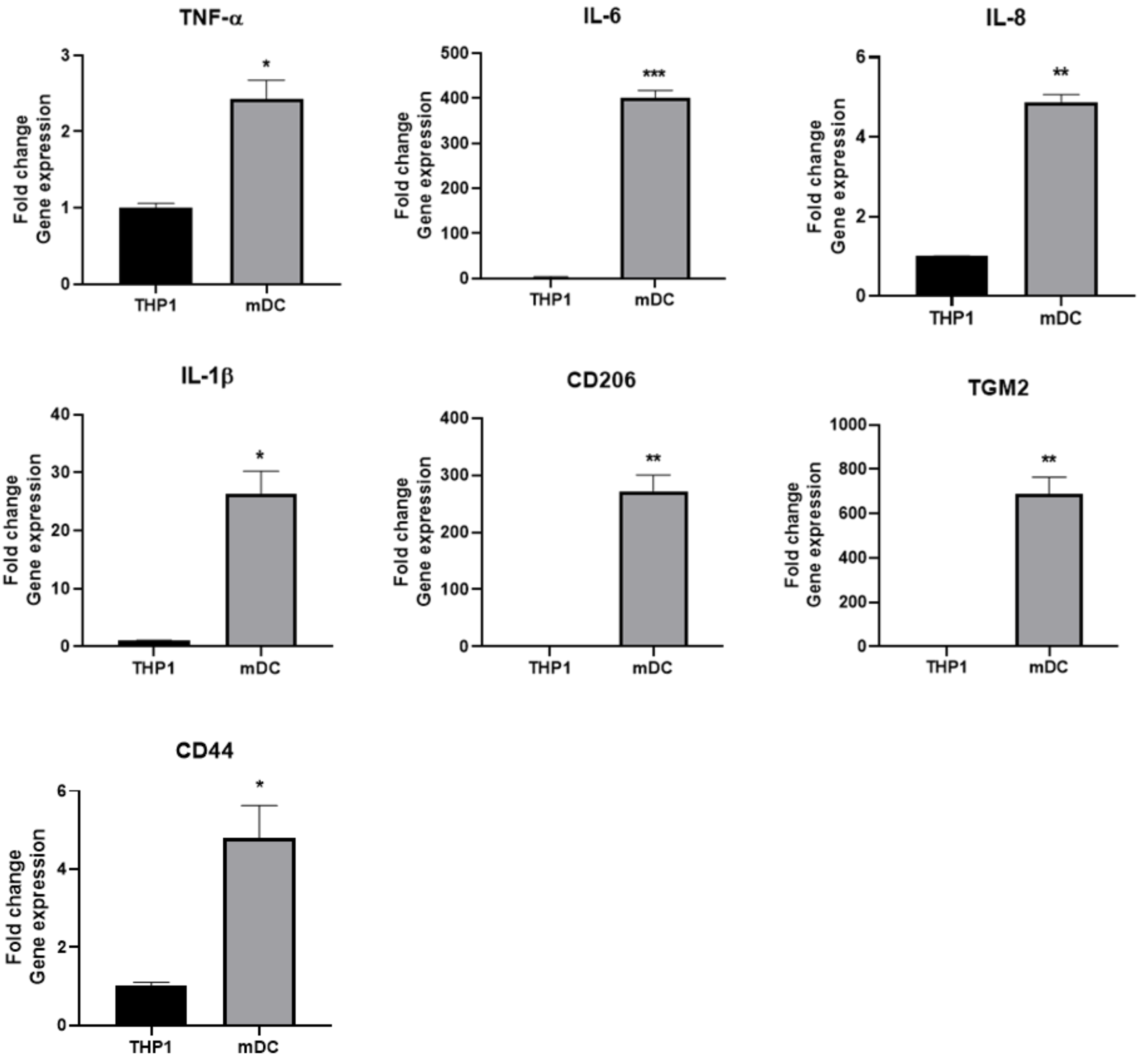

Supplementary Figure S2: Pro-inflammatory mature dendritic cell (mDC) phenotype. Mean±SD of mDC markers gene expression of TNF-α, IL-6, IL-8, IL1β, CD206, TGM2 and CD44. Genes expressions are normalized with GAPDH and TBP and data are represented in fold change. *P<0.05, **P<0.01, ***P<0.001 *vs* THP1. Data are representative of at least three independent experiments.

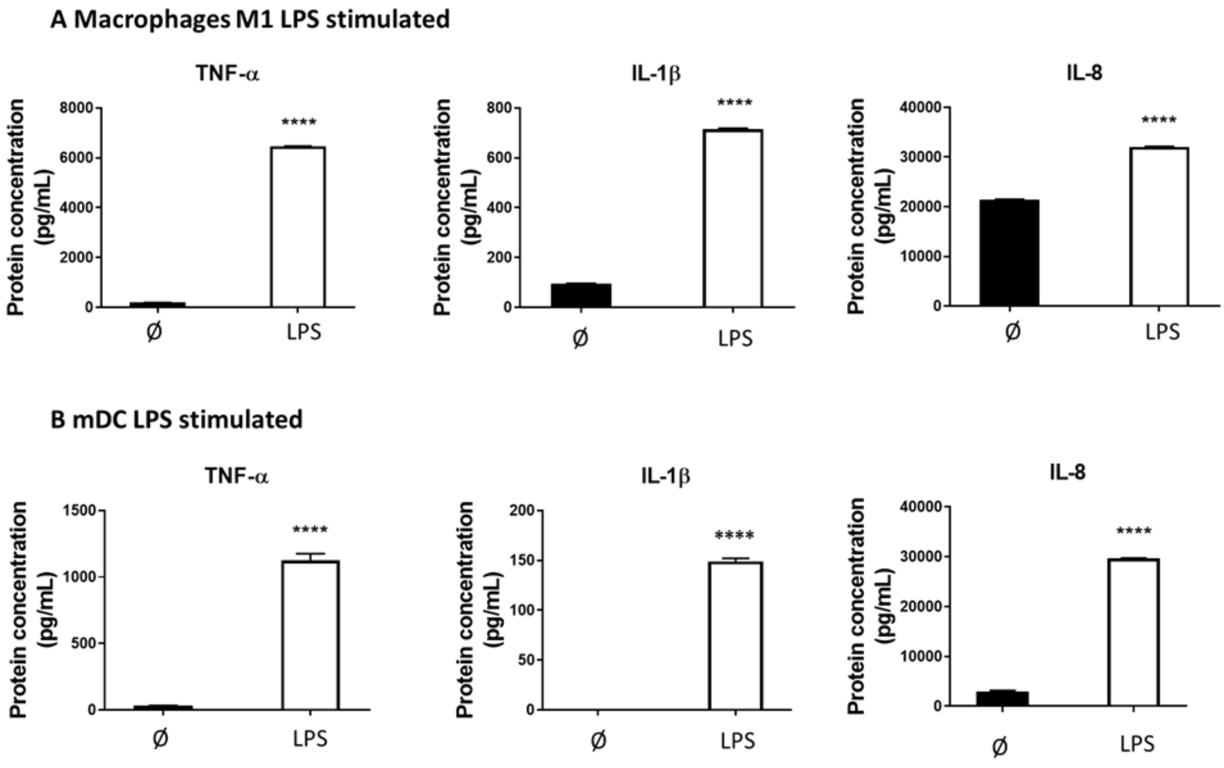

Supplementary Figure S3: LPS effect on M1 and mDC cytokine production at 24h. Supernatant produced by immune cells. Mean±SD of protein level (pg/ml) of TNF-α, IL-1β and IL-8 in macrophages M1 (A) and mDC (B) after 24h stimulation of LPS at 1µg/ml. Data are representative of at least three independent experiments. ****P<0.0001 *vs* unstimulated (Ø) immune cells.

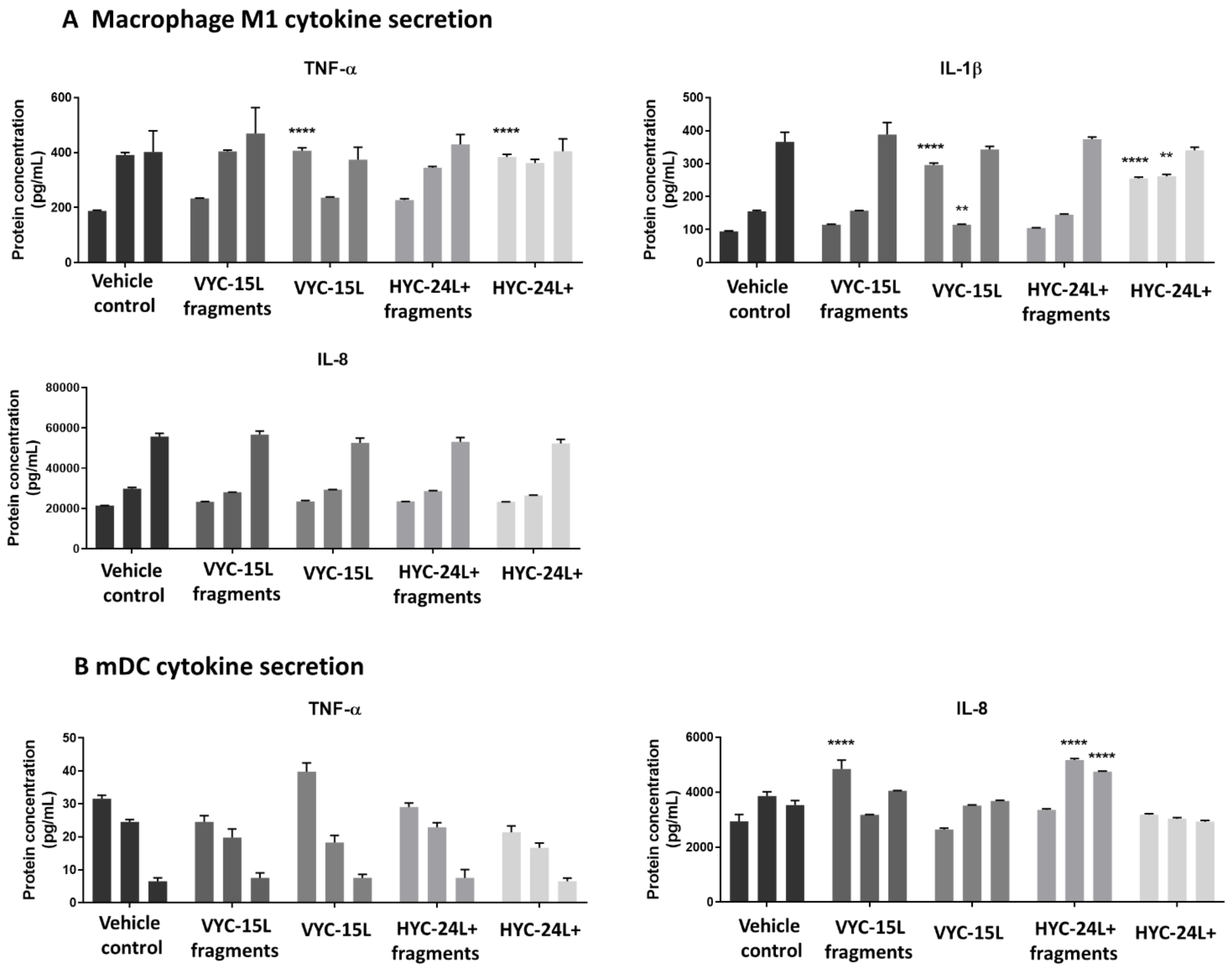

Supplementary Figure S4: Effects of HA and HA degradation products on cytokines release by M1 and mDC at the 24h, 48h and 72h time points. Mean±SD of protein level (pg/ml) of TNF-α, IL-8 and IL-1β (except for mDC), in the condition medium of M1 (A) and mDC (B) after stimulation with vehicle, VYC-15L fragments, VYC-15L, HYC-24L+ fragments and HYC-24L+. For each condition, left column corresponds to 24h, middle column corresponds to 48h and right column corresponds to 72h stimulation. Data are representative of at least three independent experiments. **$P<0.01$, ****$P<0.0001$ *vs* the respective vehicle control.

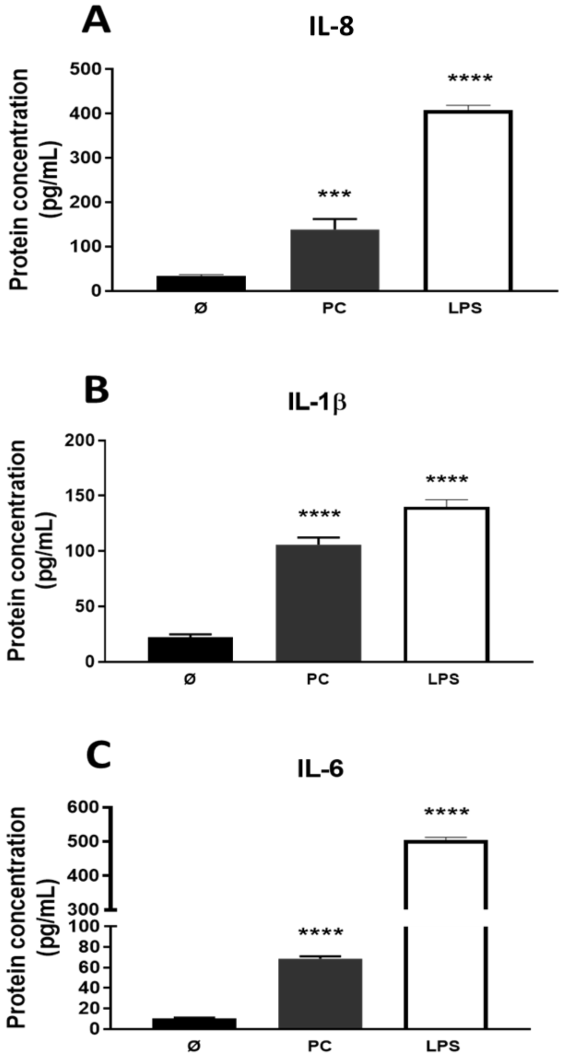

Supplementary figure S5: low-grade inflammation phenotype for preconditioned dermal fibroblasts. Cytokine release in unstimulated (Ø), preconditioned (PC) and LPS-stimulated dermal fibroblasts. Mean±SD of protein level (pg/ml) of IL-8 (A), IL-1β (B) and IL-6 (C) 72h post preconditioning. LPS is considered as the positive control for inflammation. ***$P<0.001$, ****$P<0.0001$. *vs* non-preconditioned (non-inflammatory) unstimulated fibroblast (Ø).

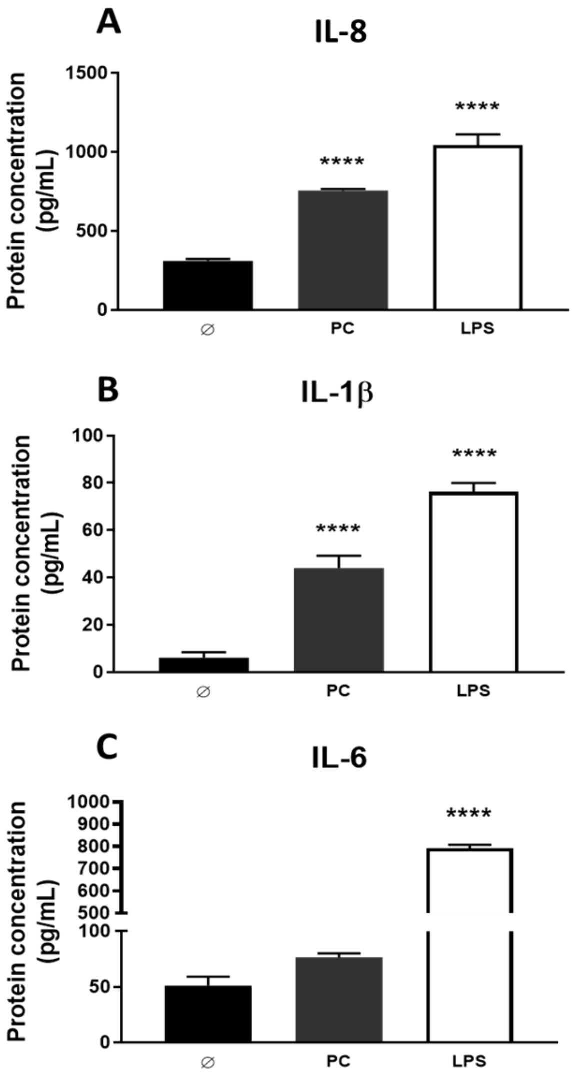

Supplementary Figure S6: low-grade inflammation phenotype for preconditioned HDMEC. Cytokine release in unstimulated (Ø), preconditioned (PC) and LPS-stimulated dermal endothelial cells (HDMEC). Mean±SD of protein level (pg/ml) of IL-8 (A), IL-1β (B) and IL-6 (C) 72h-post preconditioning. LPS is considered as positive control for inflammation. ****P<0.00001 *vs* non-preconditioned (non-inflammatory) unstimulated HDMEC (Ø).

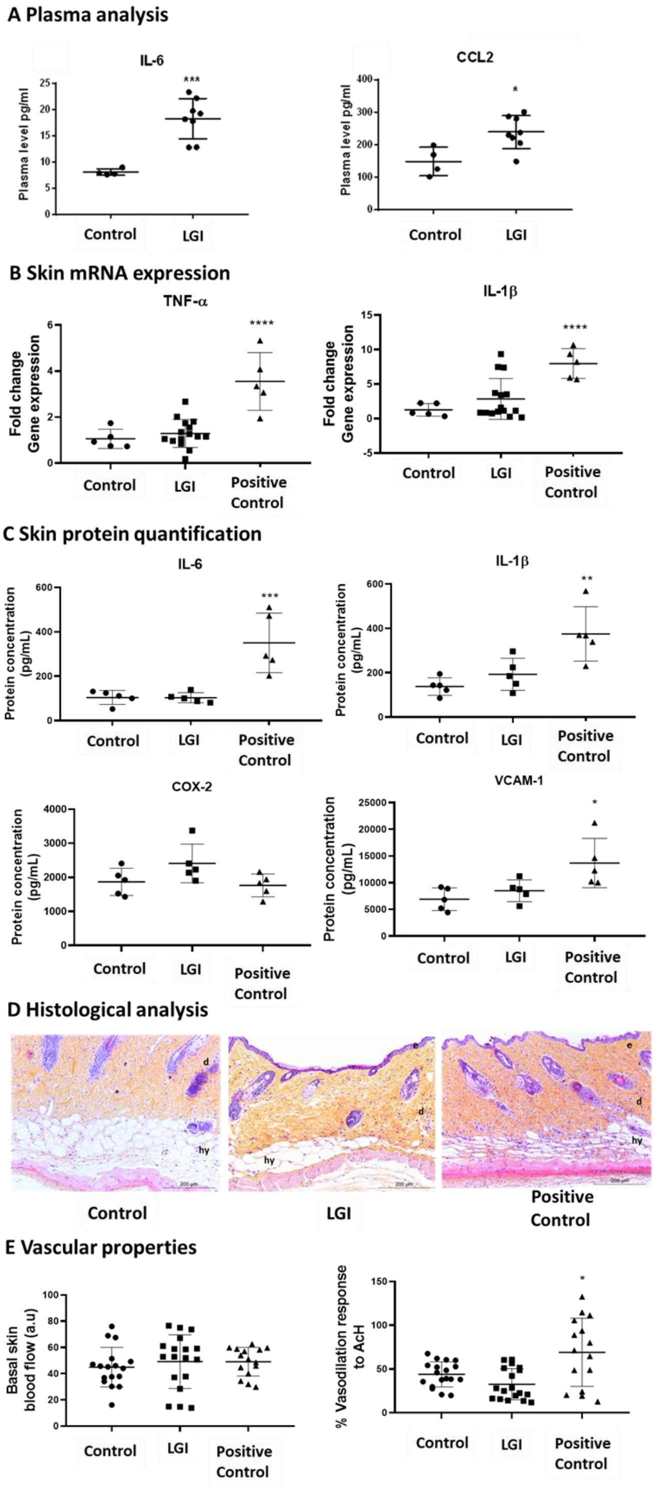

Supplementary figure S7: Phenotype of systemic low-grade inflammation induced by a chronic treatment of low dose of LPS in mice. Scatter dot plot showing: A, IL-6 and CCL2 plasma level in control mice (n=4) and in systemic low-grade inflammatory (LGI) mice (n=8); B, fold change gene expression of TNF-α and IL-1β in skin biopsy from control mice (n=5), LGI mice (n=15) and positive control mice treated with an acute intradermal injection of LPS (n=5); C, protein level of IL-6, IL-1β, COX-2 and VCAM-1 in control mice (n=5), LGI mice (n=5) and positive LPS control mice (n=5); D, representative histological analysis with HES coloration skin in one control mouse, one LGI mouse and one positive LPS control mice; E, vascular properties with basal skin blood flow and acetylcholine (ach)-mediated microvascular reactivity in control mice (n=17), LGI mice (n=18) and positive LPS control mice (n=15). *P<0.05, **P<0.01, ***P<0.001, ****P<0.0001 *vs* control mice.

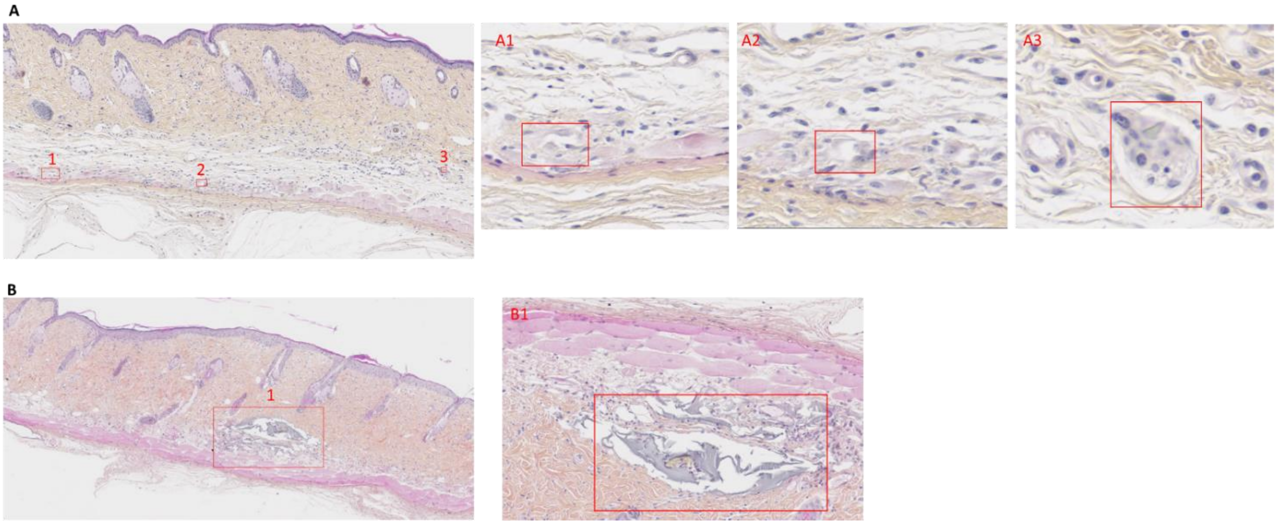

Supplementary Figure S8: Remained injected hyaluronan in the skin of low-grade inflammation (LGI) mice. Representative histological analysis with HES coloration skin in one LGI mouse 24h after an acute intradermal injection (20µl) of VYC-15L fragments (A) and HYC-24L fragments (B). Numbers indicate locations with remained injected hyaluronan, which are shown with a larger magnification on the right-hand side.
